# Statistical Approach of the Role of the Conserved CSB-PiggyBac Transposase Fusion Protein (CSB-PGBD3) in Genotype-Phenotype Correlation in Cockayne Syndrome Type B

**DOI:** 10.3389/fgene.2022.762047

**Published:** 2022-02-17

**Authors:** Rayanne Damaj-Fourcade, Nicolas Meyer, Cathy Obringer, Nicolas Le May, Nadège Calmels, Vincent Laugel

**Affiliations:** ^1^ INSERM CIC 1434, Hôpitaux Universitaires de Strasbourg, Strasbourg, France; ^2^ GMRC, Service de Santé Publique, Hôpitaux Universitaires de Strasbourg, Strasbourg, France; ^3^ ICUBE, UMR 7357, Université de Strasbourg, Illkirch, France; ^4^ Laboratoire de Génétique Médicale, Institut de Génétique Médicale d'Alsace, Faculté de Médecine de Strasbourg, Hôpitaux Universitaires de Strasbourg, Strasbourg, France; ^5^ Laboratoire de Diagnostic Génétique, Institut de Génétique Médicale d'Alsace, Nouvel Hôpital Civil, Hôpitaux Universitaires de Strasbourg, CRBS, Strasbourg, France; ^6^ Service de Pédiatrie Spécialisée et Générale, Unité de Neurologie Pédiatrique, Hôpital de Hautepierre, Hôpitaux Universitaires de Strasbourg, Strasbourg, France

**Keywords:** cockayne syndrome, CSB/ERCC6, piggyBac, clinical severity, correlation-regression analysis

## Abstract

Cockayne syndrome is a rare condition that encompasses a very wide spectrum of clinical severity. Mutations upstream of a transposon called PiggyBac Transposable Element Derived 3 in intron 5 of the *CSB/ERCC6* gene could bring about less severe forms than mutations located downstream of that transposon insertion. Our aim was to study genotype-phenotype correlation by determining whether the position of each mutation of the *CSB/ERCC6* gene has an impact on the phenotype. A hundred and forty-seven Cockayne patients, who had two pathogenic mutations in the *CSB/ERCC6* gene and for whom clinical data was available, were retrospectively selected and included in the study. Data analysis was performed under the Bayesian paradigm. Analysis of the proportion of the different subtypes of Cockayne syndrome according to the position of the mutations was done using an ordinal logistic regression model. Using a vague prior, the risk of developing a more severe subtype when exposed to 2 mutations downstream compared to 2 mutations upstream was 2.0 [0.9–4.5]. Estimations varied through the sensitivity analysis. We could reasonably conclude that a relationship between the number of downstream mutations and the Cockayne syndrome clinical expression exists but it is still difficult to give a precise estimate of this relationship. The real effect could be more complex that the one described in the initial model and other genetic factors might be taken into consideration together with the mutation site to better explain clinical variability.

## Introduction

Cockayne syndrome (CS) is an autosomal recessive disorder characterized by growth failure, developmental delay, microcephaly, sensorial alteration, cutaneous photosensitivity, dental anomalies and a recognizable facial appearance (Cockayne, 1946). Its incidence has been estimated at 1/360,000 births in Western Europe (Kleijer et al., 2008).

Several stages of severity have been described previously. They essentially depend on the age at first symptoms and on how quickly the disease develops (Natale, 2011; Laugel, 2013). In the classical form or CS type I, symptoms usually start at the end of the first year or in the second year of life. All the symptoms mentioned above can show up progressively. This type leads to premature death mainly in the second decade of life. CS type II is equivalent to severe congenital forms. Clinical signs are observed at birth, especially congenital microcephaly and cataracts. The evolution is always severe conducting to death before the end of the first decade. CS type III brings together mild juvenile forms. Symptoms are less severe and survival until adulthood is possible. Cerebro-oculo-facio-skeletal (COFS) syndrome is described as an arthrogryposis (Graham et al., 2001). It starts before birth and is associated with a major handicap and death within the first years of life.

Specific sensitivity to ultraviolet (UV) radiation of Cockayne cells has been related to defective DNA repair and transcription processes especially the nucleotide excision repair (NER) subpathway called transcription coupled-repair (TC-NER) (Mayne and Lehmann, 1982). The CS diagnosis can be suggested by clinical criteria (Nance and Berry, 1992; Laugel, 2013; Wilson et al., 2016a). However, diagnosis must be confirmed by assessing TCR Recovery of RNA Synthesis (RRS, decreased in CS) in cultured fibroblasts and searching for mutations in involved genes. Two major genes are responsible for CS: *CSA/ERCC8* and *CSB/ERCC6* (Troelstra et al., 1992; Henning et al., 1995). The *ERCC6* gene is located on chromosome 10q11 and contains 21 exons. A transposon, named PiggyBac Transposable Element Derived 3 (PGBD3), fits into intron 5 of the *CSB/ERCC6* gene. Consequently, in physiological state, the *CSB* gene generates a full-length CSB protein and a fusion protein CSB-PiggyBac that contains the first 465 amino acids of the CSB protein and the PGBD3 transposase (Newman et al., 2008). This chimeric protein is as highly conserved as CSB itself and its continued expression is able to reshape the transcriptome in cultured cells (Newman et al., 2008; Gray et al., 2012; Weiner and Gray, 2013). The effect of the chimeric protein is anticipated to be deleterious in the absence of functional CSB (absent or truncated) and may thus affect the clinical presentation in CS patients by directly altering the transcriptional program.

The large variety of symptoms and range of severity are a startling aspect of CS. Up to date, this clinical spectrum variability has not been justified by any genotype-phenotype correlation. The presence of a transposon in intron 5 of the *CSB/ERCC6* gene has led to express the following hypothesis: mutations upstream of intron 5 (coding exons 2–5) should generate neither a normal full length CSB protein nor a normal CSB-PiggyBac fusion protein, whereas mutations downstream of intron 5 (coding exons 6–21) should impact the CSB full length protein but generate a normal chimeric protein which is assumed to have a deleterious effect in the absence of CSB full length protein; mutations upstream of intron 5 could thus lead to less severe forms than mutations located downstream of that transposon insertion (Newman et al., 2008; Gray et al., 2012; Weiner and Gray, 2013). This hypothesis has already been considered as a possible therapeutic strategy with splice switching oligonucleotides to potentially reduce the disease severity (Sin et al., 2018).

Our aim was to test this hypothesis and study the genotype-phenotype correlation in a cohort of Cockayne patients to determine whether the position of the mutation on each allele of the *CSB/ERCC6* gene has an impact on the type of CS.

We also aimed to study the effect of the type of variants (protein-truncating variants (PTVs) vs Protein-altering variants (PAVs)) on the phenotype.

## Materials and Methods

### Patients

Our Strasbourg laboratory of genetics has a database for CS from which we retrospectively selected our patients. The database contained 154 Cockayne patients for whom genetic testing confirmed the diagnosis. Cockayne patients from both genders, who had two pathogenic mutations in the *CSB/ERCC6* gene, were included in the analysis. Patients who had pathogenic mutations in other genes within the NER pathway or died *in-utero* with therefore a lack of precise and reliable clinical data were excluded. Absence of clinical data was another exclusion criteria. Our database and fibroblast library (DC-2014-2222) have been registered at the French authority Commission Nationale de l’Informatique et des Libertés and authorized by the Comité de Protection des Personnes. Families had given full consent for genetic screening. In order to increase the number of patients in the main analysis, we searched for cases from the literature for whom both clinical and molecular data were available. We enrolled every publication that reported Cockayne patients. Articles had to be written in English and were eligible if they presented the details of the CSB mutations and clinical features of patients. Some of them were extracted through PubMed searches of the terms [“cockayne syndrome [MeSH Terms]” AND “phenotype [MeSH Terms]” AND “mutation [MeSH Terms]”]. We decided not to apply a restriction of publication date. The search was supplemented by our own previous bibliographic survey (Laugel et al., 2010).

### Data Collection

**FIGURE 1:**
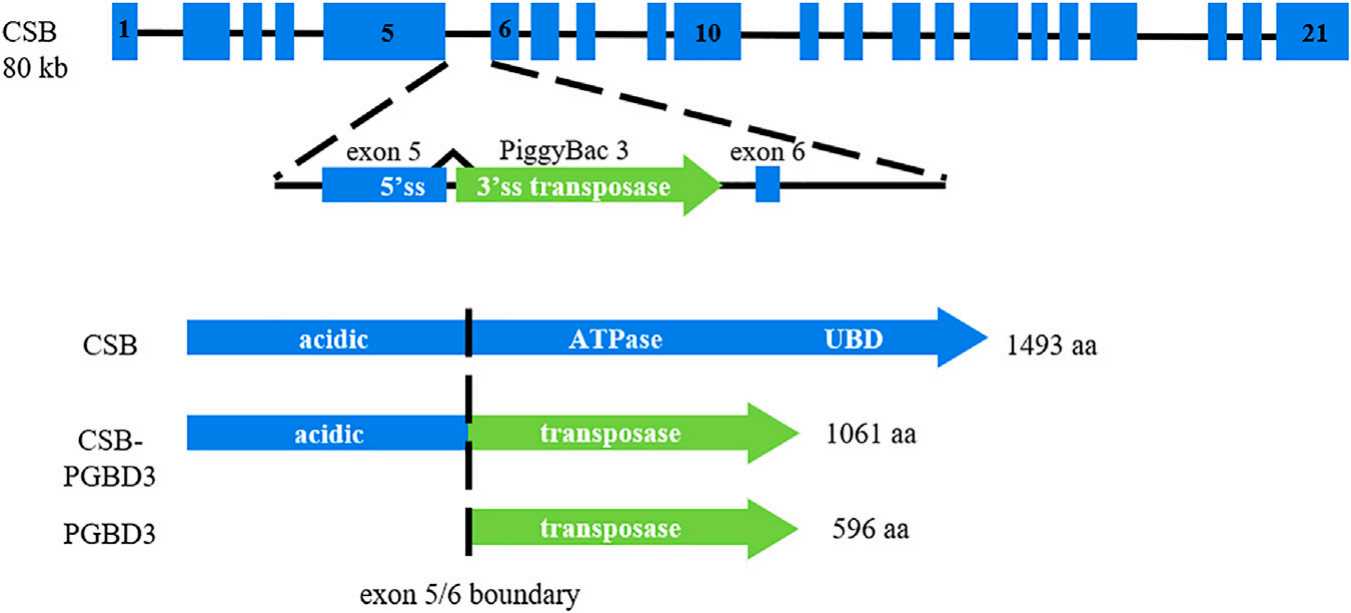
PGBD3 transposon into intron 5 of the *CSB/ERCC6* gene. Alternative splicing of CSB exons 1-5 to the PGBD3 transposase splice acceptor site enables the CSB-PGBD3 fusion protein expression. The primate CSB locus generates two other proteins: full length CSB and solitary PGBD3 transposase. Figure inspired from Gray et al. (2012).

Clinical data was collected from specific clinical files, consultation letters or hospitalization reports. It included the following variables: gender, postnatal growth parameters (height, weight, head circumference), dysmorphic signs (enophthalmia, cachexia, bird-like face), sensorial impairment characteristics (hearing loss, cataracts, retinopathy), developmental milestones, neurological symptoms (areflexia, ataxia, spasticity), intellectual disability, major cutaneous photosensitivity, arthrogryposis, age at first symptoms, age at death or last report. Genetic and molecular data contained the mutations of each patient on cDNA and their position compared with PiggyBac insertion (NM_000124.3: c.1397 + 6912, in intron 5). The position of the mutations was stratified into three groups: two mutations upstream of intron 5 (2U), two mutations downstream of intron 5 (2D), 1 mutation upstream and 1 mutation downstream of intron 5 (1U1D) (Figure 1). Clinical and genetic data of patients stemmed from literature were directly extracted from case-reports. The type of variants was stratified into two groups: PTVs and PAVs. Patients who had 1 PTV and 1 PAV were considered in the PAVs group as one of their variants had a more permissive nature.

### Patient Classification

Patients were classified into classical clinical subgroups (CS type I, CS type II, CS type III and COFS) by an expert committee without any knowledge of their mutations. The process was done according to classification routinely used (Natale, 2011; Laugel, 2013; Wilson et al., 2016a). COFS syndrome, the most severe subtype of the clinical spectrum, was defined by its own criteria: congenital microcephaly, congenital cataracts and/or microphthalmia, arthrogryposis, severe developmental delay, severe growth failure and facial dysmorphia (Laugel et al., 2008a). Furthermore, information concerning patients’ psychomotor development helped to classify them. The type II (severe CS) subgroup included patients who had their first symptoms before the age of 3 months. They could not walk and had an extremely restricted verbal communication. Type I (moderate CS) patients had generally acquired sitting position and could walk independently later on. They were able to associate words, formulate short sentences and they had good interactions with their peers. Type III (mild CS) patients could walk, run, and talk in an elaborated way. They often had learnt to read and write. For reported cases from the literature, the classification already established by the authors was taken into account except for two patients for whom classification was not mentioned in the original article (Wilson et al., 2016b). They were consequently classified by our expert committee based on available clinical data and pictures. Type II and COFS patients were finally gathered together within the same category, hereinafter referred to as the “Type II” class, as they represent the most severe phenotypes encountered.

### Statistical Analysis

Qualitative variables were described as counts and percentages. Age was described with mean (standard deviation, minimum, maximum).

Data analyses were performed under the Bayesian paradigm. Bayesian methods were used for their internal logical consistency for probabilistic inference and their ability to explicitly include previous data and expert knowledge in the data analysis (Dunson, 2001). Results are all expressed with their respective 95% credibility interval (CI) on posterior distribution (Ferreira et al., 2020). The major part of the parameters was estimated using Markov chain Monte Carlo (McMC) algorithms. A burn-in of 5000 iterations, followed by 150 000 iterations with a thinning of 3, was used for the three chains. McMC chain convergence was assessed and checked graphically.

The relationship between the position of the mutations and the type of CS was studied first using a uniform Dirichlet prior distribution Di (1, … , 1) and a Jeffreys prior Di (0.5, … , 0.5). Based on the contingency table obtained with the Jeffreys prior, Odds Ratios (OR) were estimated comparing subtypes and classes of mutations two by two. Next, analysis of the proportion of the different types of CS according to the position of the mutations in the *CSB/ERCC6* gene was done using an ordinal logistic regression model under the assumption that the disease severity would increase with the number of downstream mutations. Prior distributions were defined before conducting the analyses. A sensitivity analysis was led. As part of it, we used three different Gaussian N (mean, variance) priors for parameters of the logistic regression. A low information prior (log (OR)∼N (µ = 0, σ^2^ = 1,000)) was first used. A more precise prior (log (OR)∼N (µ = 0, σ^2^ = 0.674)) specified an OR a priori between 0.2 and 5. An optimistic prior (log (OR)∼N (µ = 1.099, σ^2^ = 0.377)) specified an OR a priori between 0.9 and 10. In this study, Bayesian results gave the probability that the OR of a severe phenotype would be greater than 1. The effect was judged relevant when the probability that the OR exceeded 1 (Pr(OR>1)) was larger than 95%. This probability should not be confused with the frequentist *p-value*.

Analysis of the proportion of the different types of CS according to the type of variants was done using an ordinal logistic regression model. As part of the sensitivity analysis, we used two different Gaussian priors for parameters which were a low information prior (log (OR)∼N (µ = 0, σ^2^ = 1,000)) and a more precise prior (log (OR)∼N (µ = 0.661, σ^2^ = 0.234)) that specified an OR a priori between 0.75 and 5. The type of variants was then included as a covariable in a multivariate model.

Our secondary outcome, age at onset according to the position of the mutations, was analyzed with a categorical predictor linear model, akin to an ANOVA model. In this model, the parameter prior distribution was N (0, 100).

All analyses were done using JAGS 4.3.0 (rjags, RRID:SCR_017573) and R 4.0.3 (R Project for Statistical Computing, RRID:SCR_001905) softwares, with all the required packages in their latest version at the time of analysis.

## Results

In the Strasbourg database, 154 Cockayne patients were available. Patients who had pathogenic mutations in *ERCC1* (n = 2), *ERCC2* (n = 2), *ERCC3* (n = 1) and *CSA/ERCC8* (n = 51) genes were excluded. Six patients died *in-utero* and were also excluded from the analysis. Clinical data was not available for 7 patients. Eighty-five patients from the Strasbourg database finally met the inclusion criteria to which 62 case-reports from the literature were added (Brumback et al., 1978; Troelstra et al., 1992; Lehmann et al., 1993; Stefanini et al., 1996; Mallery et al., 1998; Colella et al., 1999; Meira et al., 2000; Falik-Zaccai et al., 2008; Hashimoto et al., 2008; Wilson et al., 2016b; Kou et al., 2018; Sanchez-Roman et al., 2018). A total of 147 patients were included in the study, of whom 24 (16.3%) had 2U of PiggyBac, 18 (12.2%) had 1U1D of PiggyBac and 105 (71.4%) had 2D of PiggyBac (Table 1). The mean age at first symptoms was 8 months (standard error = 13.4). The values ranged from 0 (birth, minimum) to 72 months (maximum). This variable was missing for 24 patients.

**TABLE 1:** Patient characteristics.

	Entire cohort (n = 147)		Strasbourg database (n = 85)		Case-reports (n = 62)	
Characteristics	No	%^6^	No	%^6^	No	%^6^
Sex						
Male	80	58.4	50	59.5	30	56.6
Female	57	41.6	34	40.5	23	43.4
NA^1^	10		1		9	
Alleles						
Homozygous	88	59.9	53	62.4	35	56.5
Heterozygous	59	40.1	32	37.6	27	43.5
Position of the mutations^2^						
2 upstream	24	16.3	13	15.3	11	17.7
1 upstream/1	18	12.2	9	10.6	9	14.5
downstream
2 downstream	105	71.4	63	74.1	42	67.7
Fusion protein^3^						
Absent	24	16.3	13	15.3	11	17.7
Present	123	83.7	72	84.7	51	82.3
Subtype						
I	54	36.7	31	36.5	23	37.1
I or II^4^	4	2.7	4	4.7		
I/II^5^	6	4.1	6	7.1		
II	71	48.3	36	42.4	35	56.4
III	12	8.2	8	9.4	4	6.5

1 .NA, not available.

2 .Regarding to PiggyBac insertion.

3 .Prediction made according to the position of the mutations.

4 .Patients who could be either in subtype I or subtype II.

5 .Patients who are at the boundary between subtypes I and II.

6 .Proportions were calculated without taking into account NA data.

Four patients could either correspond to a type I or a type II and six patients were at the boundary between type I and type II. Those 10 patients were therefore not included in the ordinal logistic regression model. Another patient was also removed from this analysis because he could not be classified according to the current criteria.

**FIGURE 2:**
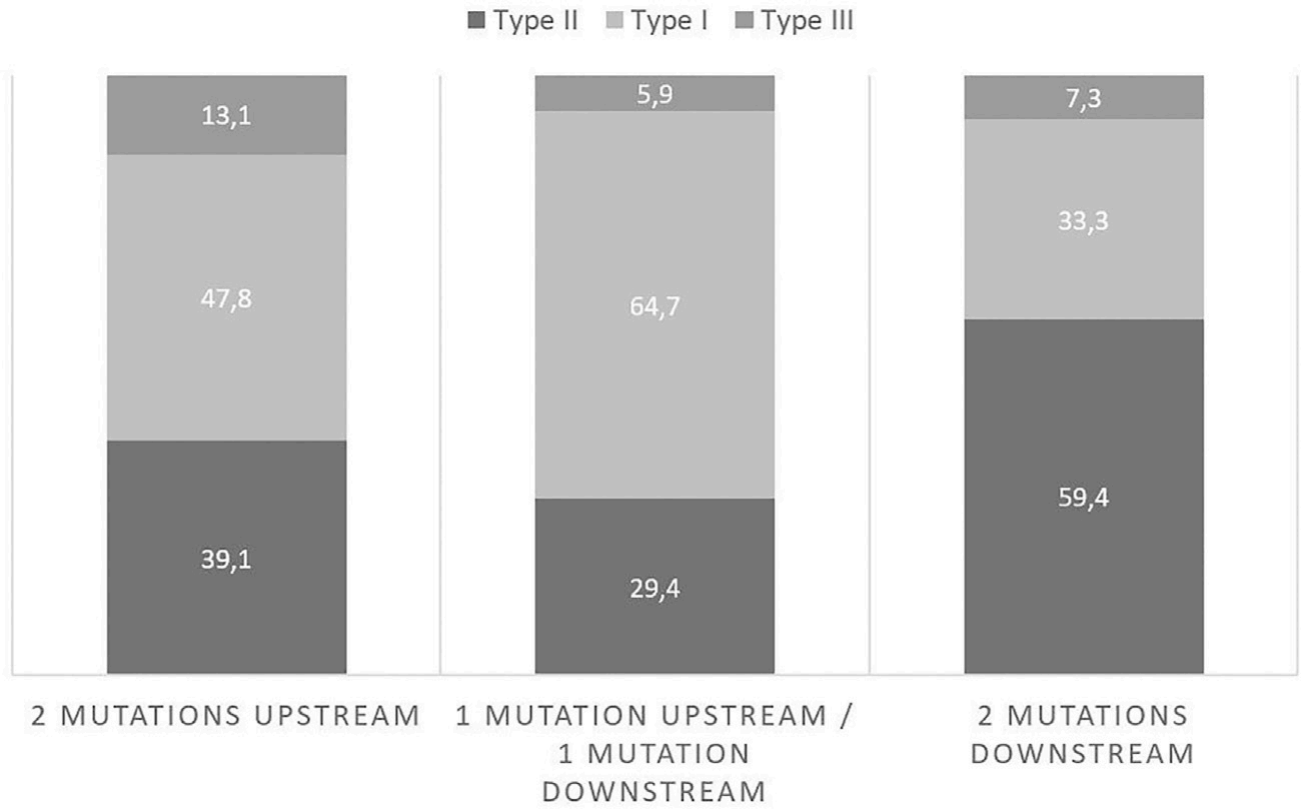
Proportions of subtypes in each mutation group.

The proportions of patients in each subtype/mutations group of the cohort are presented in Table 2. Looking closer to the proportions of patients in each mutation group (Figure 2), there were more type II patients in the 2D group than in the 2U group and the 1U1D group (59.4 vs 39.1% and 29.4% respectively).

**TABLE 2:** Numbers (%) of patients in each subtype/mutations group.

	Type II	Type I	Type III	Total
2U	9 (6.7)	11 (8.1)	3 (2.2)	23 (17.0)
1U1D	5 (3.7)	11 (8.1)	1 (0.7)	17 (12.5)
2D	57 (41.9)	32 (23.5)	7 (5.1)	96 (70.5)
Total	71 (52.3)	54 (39.7)	11 (8)	136 (100)

The first analysis, using a Dirichlet distribution with a Jeffreys prior, showed the following results (Table 3). The risk of developing type II disease as compared to type I was increased by 3.9 (CI: [1.3–12.8]) for patients having 2D in comparison to 1U1D. Patients exposed to 2D in comparison to 2U had a risk multiplicated by 2.2 (CI: [0.8–5.9]) to develop a type II as compared to a type I and a risk multiplicated by 2.8 (CI: [0.6–12.0]) to develop a type II as compared to a type III. Finally, patients exposed to 2D in comparison to those exposed to at least one mutation upstream (2U and 1U1D) had 2.8 (CI: [1.3–6.3]) more risk to have a type II compared to a type I with a Pr(OR>1) reaching 99.5%.

**TABLE 3:** Risk of having a subtype compared to another depending on the position of the mutations.

	Subtype II vs I	Subtype I vs III	Subtype II vs III
2D vs 1U1D	^a^3.9 [1.3–12.8]; 99.3	0.5 [0.0–2.8]; 22.2	1.8 [0.1–12.6]; 70.5
1U1D vs 2U	0.6 [0.1–2.2]; 20.2	2.7 [0.3–38.8]; 81.8	1.5 [0.2–23.1]; 63.9
2D vs 2U	2.2 [0.8–5.9]; 94.1	1.3 [0.3–5.4]; 62.4	2.8 [0.6–12.0]; 90.1

a .Odds ratios [OR] are presented with their credibility intervals [CI] and probabilities of being higher than 1 (%).

Ordinal logistic regression using a vague prior (N (0, 1,000)) showed that the risk of having a more severe subtype when exposed to 2D compared to 2U was 2.0 (CI: [0.9–4.5]) with a Pr(OR>1) of 94.1%. That risk when exposed to 1U1D compared to 2U, was of 0.9 (CI [0.3–2.7]) with a Pr(OR>1) equal to 42.6%. Results obtained with the two other priors are presented in Table 4. Estimations varied through the sensitivity analysis.

**TABLE 4:** Ordinal logistic regression odds estimates. Risk of having a more severe subtype depending on the position of the mutations with three different priors. OR, odds ratio; CI, credibility intervals; Pr(OR>1), probability that the OR is higher than 1 (%). 1: OR_p_ [CI], odds ratio *a priori* with its credibility interval used to determine prior distribution for the model parameters.

Priors N (μ, σ^2^)	Mutations	OR [CI]; Pr(OR>1)
Log (OR)∼N (0, 1,000)	1U1D	0.9 [0.3–2.7]; 42.6
OR_p_ [CI]^1^ = 1 [0–8.3*10^26^]	2D	2.0 [0.9–4.5]; 94.1
	2U	Reference
Log (OR)∼N (0, 0.674)	1U1D	0.9 [0.4–2.1]; 37.0
OR_p_ [CI]^1^ = 1 [0.2–5]	2D	1.7 [0.9–3.5]; 93.6
	2U	Reference
Log (OR)∼N (1.099, 0.377)	1U1D	1.6 [0.7–3.5]; 87.8
OR_p_ [CI]^1^ = 3.0 [0.9–10]	2D	2.6 [1.4–5.0]; 99.8
	2U	Reference

Results of the model used to estimate age at first symptoms depending on the mutation position were as follows. Patients with 2U had a mean age at first symptoms of 11.8 [6.7–16.9] months which was higher than the mean age of patients with 2D. Indeed, those ones had 6.5 [3.8–9.3] months at first symptoms. The probability of a difference (Pr (diff) > 0) between 2D and 2U patients reached 96.2%. Patients with 1U1D had a mean age of 11.4 [5.1–17.6] months when first symptoms appeared. Pr (diff) > 0 were of 91.7 and 54.1% comparing respectively 2D to 1U1D and 1U1D to 2U.

We then looked at the potential effect of the mutation type (PAV or PTV). The univariate model aiming to study the effect of the type of variants on the phenotype included 132 patients which corresponded to complete cases. Using a vague prior (N (0, 1,000)), ordinal logistic regression pointed out that the risk of having a more severe subtype with PTVs as compared to PAVs was 0.8 (CI [0.4–1.6]) with a Pr(OR>1) of 25.1%. Results obtained with the prior in favour of the hypothesis that PTVs could induce more severe phenotypes (N (0.661, 0.234)) showed a risk of developing a severe phenotype multiplicated by 1.1 (CI [0.6–1.9]) with PTVs as compared to PAVs with a Pr (OR>1) of 60.1%.

The multivariate ordinal logistic regressions (Supplementary Material) which included the position and the type of variants as covariates did not change the conclusions that we could make with univariate models as OR and their CI did not fundamentally vary even through the sensitivity analysis that we conducted. Both analyses were unable to show a clear relationship between the mutation type and the CS phenotype.

However, PAVs and PTVs do not seem to be randomly distributed on either side of the PiggyBac insertion and this could have biased the previous analyses, as no PAV could be identified upstream of the PiggyBac insertion. Strikingly, all 2U patients showed only PTVs (23/23), whereas in the 2D group only 55/92 patients were classified in the PTV subgroup. In the 2U group, 14/23 (61%) patients with PTVs had a moderate or mild phenotype, as compared to 24/55 (44%) for the PTVs patients in the 2D group. In the 1U1D group, the upstream mutation was always a PTV. When considering only the 2D group, the distribution of the clinical subgroups was similar between the PAVs and PTVs groups, respectively 64.9% of type II/COFS, 27% of type I, 8.1% of type III with PAVs and 56.4, 36.3, 7.3% with PTVs. The complete distribution of PAVs and PTVs is displayed in Supplementary Material.

## Discussion

CS encompasses a large spectrum of clinical presentations from the most severe prenatal subtype to the adult-onset subtype. Accumulating clinical data shows that Cockayne syndrome is actually a continuous spectrum of forms of varying severity (Laugel, 2013) and no study has brought to light a clear-cut genotype-phenotype correlation so far. Horibata and others (Horibata et al., 2004) first suggested that CSB truncations generating no functional protein resulted in the mild phenotype of UV sensitive syndrome, whereas more C-terminal truncations might generate inactive protein that could interfere with other processes, thereby resulting in more severe phenotypes. Consistently, Hashimoto *et al* also reported a mild CS phenotype linked to an early truncating mutation (Hashimoto et al., 2008). It led to the “PiggyBac hypothesis” stating that early truncating mutations upstream of the PiggyBac insertion in intron 5 (i.e., upstream of residue 466 in the protein sequence) could be paradoxically associated with the mildest phenotypes and that the CSB-PiggyBac fusion protein could have a deleterious effect in the absence of the full length CSB protein (Newman et al., 2008; Gray et al., 2012; Weiner and Gray, 2013). The evolutionary conservation of the CSB-PiggyBac fusion protein could further substantiate the fact that it retains a biological effect and experimental data has shown that the fusion protein could indeed inhibits TCR of oxidative damage but facilitates TCR of UV damage (Bailey et al., 2012). However, later reports showed that this hypothesis could not fully account for all reported CS cases especially in some particular genetics isolates with high consanguinity (Laugel et al., 2008b; Falik-Zaccai et al., 2008).

By applying Bayesian ordinal logistic regression with a very low informative prior, we identified that the risk of having a severe phenotype was increased by 2.0 (CI: [0.9–4.5]) in patients with 2D in comparison to patients with 2U. Furthermore, with a prior slightly in favour of our hypothesis (Table 4), the point estimate was larger (2.6 [1.4–5.0]) and Pr(OR>1) reached 99.8%. These results do show a tendency in the expected direction and we could exclude the absence of effect which would have been the case if the OR had been estimated at 1 with a narrow credible interval.

Despite having this high probability of the OR being higher than 1 and a tendency to more severe features when presenting 2D, estimations were quite sensitive to prior choice. A residual uncertainty in the estimates remains which can be due either to the small amount of data available or to a small amount of prior knowledge on the subject, because of a lack of a genetic model to substantiate the relationship. Other genetic factors might, together with the site of the mutations, explain better the variability of the disease severity.

Comparing 1U1D to 2U with the low informative prior, the risk of having a severe clinical presentation was 0.9 [0.3–2.7] times higher. This result was surprising considering that the fusion protein is supposed deleterious in the absence of full length CSB and we would have therefore expected an increased risk of severe phenotype when having at least one mutation downstream of PiggyBac. An optimistic prior slightly modified these results, leading to a risk of developing a severe phenotype increased to 1.6 [0.7–3.5]. In the light of these results, we could not conclude to an effect of 1U1D on severe phenotypes. A rather important level of uncertainty remains. Estimation of the OR when exposed to 2D in comparison to 1U1D showed a risk increased by 3.9 (CI: [1.3–12.8]; Pr(OR>1) = 99.3%) to develop a type II compared to a type I. We would not have thought, *a priori*, to find such an increased risk when comparing those two mutations classes because of expected protein fusion expression for patients having either 1U1D or 2D. Comparing mean age at first symptoms showed that 1U1D patients had a mean age closer to 2U patients than to 2D patients although all CI overlapped. We chose this variable as secondary outcome because age at first symptoms is an information that contributes to ascertain classification and reflects the disease degree of severity. The fact that the 1U1D patients behave in a similar manner as 2U patients, combined with the observation that all upstream mutations are PTVs, might suggest that the deleterious effect of the CSB-PiggyBac fusion protein might have a quantitative threshold and that one allele expressing the fusion protein is not enough to see this deleterious effect. These findings raised a question that would need further experiments to be answered. We could wonder if patients with 1U1D are more similar to 2U patients or to 2D patients taking into consideration other potential explanative factors.

To sum up, our statistical approach can reasonably confirm a relationship between the position of the mutations on either side of the PiggyBac insertion and the clinical severity. Our data suggest a protective effect when there is at least one mutation upstream of this insertion model and that upstream mutations are all truncating mutations. It is however still difficult to give a precise estimate of this effect but our data suggest that other factors are probably at play.

Other genetic factors should be considered together with the position of the mutations in the understanding of genotype-phenotype correlation. In CS-A patients bearing homozygous mutations, it has been shown previously that missense mutations appear to be more frequently associated with mild phenotypes than protein-truncating mutations (Calmels et al., 2018). Although there are fewer missense mutations than protein-truncating mutations in CS-B patients, the severity of the clinical features might also be partly related to the mutation type in CSB patients. Previous results, comparing splice site mutations to truncating mutations ground the argument that mutation type should be considered (Schalk, 2018). In this work, median severity score for CSA and CSB patients was 7 (confidence interval [3–12]) for patients having 2 splice site mutations whereas it lowered to 4 (confidence interval [0–8]) for patients with 2 truncating mutations. Splice site mutations were associated with less severe phenotypes than truncating mutations. This could be explained by the permissive nature of splice site mutations allowing synthesis of a small amount of residual normal protein. In the present study, the results of the models including PTVs/PAVs do not permit to conclude neither to the presence nor the absence of a global effect of the type of variants. This might be due to more complex and combined effects between different factors and we have observed that the analysis of the PTVs/PAVs impact on the phenotype is clearly biased by the fact that all upstream mutations are PTVs. Indeed, our results suggest that only truncating mutations can be pathogenic when located upstream of the PiggyBac insertion: this might be due to the impact of upstream mutations on the CSB full length protein or on the CSB-PiggyBac fusion protein or on both proteins. On the other hand, when considering only patients with downstream mutations (2D group), the distribution of the clinical profiles was very similar in PTVs and PAVs patients, which was not in favour of a more deleterious effect of truncating mutations downstream of the PiggyBac insertion. More data are needed to clarify the role of the type of mutations in combination with the impact of the position.

In xeroderma pigmentosum (XP) group D gene (*XPD/ERCC2*), Ueda *et al* showed that a given mutation affects specific molecular processes during transcription (Ueda et al., 2009). As a result, different biochemical phenotypes are observed for each *XPD* mutation. They supported Andressoo’s biallelic hypothesis in which both alleles contribute to the phenotype (Andressoo et al., 2006). Considering this, it would be interesting to investigate the role of the second mutation in compound heterozygous CS-B patients who have a common mutation and to see if it has, as suggested in XP patients, a major role in determining the different clinical symptoms. Comparing phenotypes between homozygous and heterozygous patients with a shared mutation and both mutations, either upstream or downstream of PiggyBac, might also be an opportunity to better understand the role of genetic effects on phenotypes.

The main strength of our study is that, to the best of our knowledge, it was the first to include as many Cockayne patients, considering the disease rarity, to study the impact of the mutation position on the CS phenotype. Moreover, the statistical approach used herein to address this problem had not been used so far. A limitation of our study is, from a statistical point of view, the rarity of the disease and the number of patients included, which hindered a more precise assessment of the statistical relationship, despite the use of reasonably informative prior parameter estimates in our models. Prospective western blot studies would also be needed to further assess the actual presence of the CSB protein, of the PiggyBac transposase and of the chimeric PiggyBac-CSB protein in patient samples to confirm the statistical prediction. Due to difficulties to determine which type of CS affects a patient, an overlap of clinical presentations as seen in our cohort, lack of thresholds between groups and potential progression within a group, clinicians from our team recently developed a severity score (Spitz et al., 2021). Using this score in the future could help to study genotype-phenotype correlation since it allows a repeated assessment through all the years of follow-up. Quantitative nature of this assessment seems to fit well to the continuous spectrum described earlier and may represent in a more accurate and reliable way CS evolution of severity.

In conclusion, our statistical approach confirms a likely deleterious effect of the PiggyBac-CSB chimeric protein in the absence of a normal full length CSB protein and that this effect participates in the resulting clinical presentation. Our results also suggest that this is probably not the only factor involved in genotype-phenotype correlation as we would then have expected a more notable effect. We still need more data to get a precise estimation of the risk associated with a downstream mutation and the potential combining effect of the type of mutation. Further data should be collected and added to the present study to reach a better understanding of genotype-phenotype correlations for CSB.

## Data Availability

The data analyzed in this study is subject to the following licenses/restrictions: The datasets used and analyzed during the current study are available from the corresponding author on reasonable request. Requests to access these datasets should be directed to VL, vincent.laugel@chru-strasbourg.fr.

## Abbreviations

CS, Cockayne syndrome; RRS, recovery of RNA synthesis; OR, odds ratio; 2U, 2 mutations upstream of PiggyBac; 1U1D, 1 mutation upstream and 1 mutation downstream of PiggyBac; 2D, 2 mutations downstream of PiggyBac.
