# Supplementary material for: Statistical Approach of the Role of the Conserved CSB-PiggyBac Transposase Fusion Protein (CSB-PGBD3) in Genotype-Phenotype Correlation in Cockayne Syndrome Type B
Source: Front Genet. 2022 Feb 17;13:762047. doi: 10.3389/fgene.2022.762047 (PMC8891132; doi:10.3389/fgene.2022.762047)
Supplement: Supplementary file 1 [file DataSheet1.PDF]

# *Supplementary Material*

**Suppl Table 1.** Clinical description by type of Cockayne syndrome. 1: NA = not available; 2: IUGR / lbw = intrauterine growth retardation / low birth weight; 3: proportions were calculated without taking into account NA data.

| | Type II | | Type I | | Type III | |
|---|---|---|---|---|---|---|
| **Clinical data** | **No.** | **%[3]** | **No.** | **%[3]** | **No.** | **%[3]** |
| **Weight failure** | | | | | | |
| Yes | 68 | 100 | 51 | 98.1 | 10 | 100 |
| No | 0 | 0 | 1 | 1.9 | 0 | 0 |
| NA[1] | 3 | | 2 | | 1 | |
| **Height failure** | | | | | | |
| Yes | 67 | 98.5 | 48 | 96 | 10 | 100 |
| No | 1 | 1.5 | 2 | 4 | 0 | 0 |
| NA[1] | 3 | | 4 | | 1 | |
| **IUGR / lbw[2]** | | | | | | |
| Yes | 44 | 78.6 | 7 | 20.6 | 1 | 12.5 |
| No | 12 | 21.4 | 27 | 79.4 | 7 | 87.5 |
| NA[1] | 15 | | 20 | | 3 | |
| **Facial dysmorphia** | | | | | | |
| Yes | 49 | 84.5 | 44 | 91.7 | 9 | 81.8 |
| No | 9 | 15.5 | 4 | 8.3 | 2 | 18.2 |
| NA[1] | 13 | | 6 | | 0 | |
| **Intellectual disability** | | | | | | |
| Yes | 62 | 98.4 | 46 | 95.8 | 9 | 90 |
| No | 1 | 1.6 | 2 | 4.2 | 1 | 10 |
| NA[1] | 8 | | 6 | | 1 | |
| **Microcephaly** | | | | | | |
| Yes | 65 | 100 | 43 | 95.6 | 8 | 88.9 |
| No | 0 | 0 | 2 | 4.4 | 1 | 11.1 |
| NA[1] | 6 | | 9 | | 2 | |
| **Cataracts** | | | | | | |
| Yes | 57 | 89.1 | 12 | 30 | 2 | 28.6 |
| No | 7 | 10.9 | 28 | 70 | 5 | 71.4 |
| NA[1] | 7 | | 14 | | 4 | |
| **Microphthalmia** | | | | | | |
| Yes | 24 | 60 | 1 | 4.2 | 1 | 14.3 |
| No | 16 | 40 | 23 | 95.8 | 6 | 85.7 |
| NA[1] | 31 | | 30 | | 4 | |
| **Retinal degeneration** | | | | | | |
| Yes | 24 | 58.5 | 25 | 58.1 | 4 | 50 |
| No | 17 | 41.5 | 18 | 41.9 | 4 | 50 |
| NA[1] | 30 | | 11 | | 3 | |
| **Deafness** | | | | | | |

| | | | | | | |
|---|---|---|---|---|---|---|
| Yes | 34 | 73.9 | 25 | 56.8 | 6 | 75 |
| No | 12 | 26.1 | 19 | 43.2 | 2 | 25 |
| NA[1] | 25 | | 10 | | 3 | |
| **Cutaneous photosensitivity** | | | | | | |
| Yes | 33 | 61.1 | 39 | 84.8 | 6 | 66.7 |
| No | 21 | 38.9 | 7 | 15.2 | 3 | 33.3 |
| NA[1] | 17 | | 8 | | 2 | |
| **Dental anomalies** | | | | | | |
| Yes | 20 | 57.1 | 17 | 53.1 | 4 | 57.1 |
| No | 15 | 42.9 | 15 | 46.9 | 3 | 42.9 |
| NA[1] | 36 | | 22 | | 4 | |
| **Arthrogryposis** | | | | | | |
| Yes | 18 | 40 | 1 | 3.4 | 0 | 0 |
| No | 27 | 60 | 28 | 96.6 | 8 | 100 |
| NA[1] | 26 | | 25 | | 3 | |

**Suppl Table 2.** Clinical description by the position of the mutations. 1: NA = not available; 2: IUGR / lbw = intrauterine growth retardation / low birth weight; 3: proportions were calculated without taking into account NA data; 4: 2 mutations upstream of PiggyBac; 5: 1 mutation upstream and 1 mutation downstream of PiggyBac; 6: 2 mutations downstream of PiggyBac.

| | 2U[4] | | 1U1D[5] | | 2D[6] | |
|---|---|---|---|---|---|---|
| **Clinical data** | **No.** | **%[3]** | **No.** | **%[3]** | **No.** | **%[3]** |
| **Weight failure** | | | | | | |
| Yes | 22 | 95.7 | 17 | 100 | 90 | 100 |
| No | 1 | 4.3 | 0 | 0 | 0 | 0 |
| NA[1] | 0 | | 0 | | 6 | |
| **Height failure** | | | | | | |
| Yes | 22 | 95.7 | 17 | 100 | 86 | 97.7 |
| No | 1 | 4.3 | 0 | 0 | 2 | 2.3 |
| NA[1] | 0 | | 0 | | 8 | |
| **IUGR / lbw[2]** | | | | | | |
| Yes | 8 | 36.4 | 3 | 30 | 41 | 62.1 |
| No | 14 | 63.6 | 7 | 70 | 25 | 37.9 |
| NA[1] | 1 | | 7 | | 30 | |
| **Facial dysmorphia** | | | | | | |
| Yes | 19 | 90.5 | 14 | 93.3 | 69 | 85.2 |
| No | 2 | 9.5 | 1 | 6.7 | 12 | 14.8 |
| NA[1] | 2 | | 2 | | 15 | |
| **Intellectual disability** | | | | | | |
| Yes | 22 | 100 | 16 | 94.1 | 79 | 96.3 |
| No | 0 | 0 | 1 | 5.9 | 3 | 3.7 |
| NA[1] | 1 | | 0 | | 14 | |
| **Microcephaly** | | | | | | |
| Yes | 21 | 95.5 | 14 | 93.3 | 81 | 98.8 |
| No | 1 | 4.5 | 1 | 6.7 | 1 | 1.2 |
| NA[1] | 1 | | 2 | | 14 | |
| **Cataracts** | | | | | | |
| Yes | 9 | 45 | 10 | 76.9 | 52 | 66.7 |
| No | 11 | 55 | 3 | 23.1 | 26 | 33.3 |
| NA[1] | 3 | | 4 | | 18 | |
| **Microphthalmia** | | | | | | |
| Yes | 3 | 5.3 | 2 | 22.2 | 21 | 48.8 |
| No | 16 | 94.7 | 7 | 77.8 | 22 | 51.2 |
| NA[1] | 4 | | 8 | | 53 | |
| **Retinal degeneration** | | | | | | |
| Yes | 7 | 36.8 | 8 | 66.7 | 38 | 62.3 |
| No | 12 | 63.2 | 4 | 33.3 | 23 | 37.7 |
| NA[1] | 4 | | 5 | | 35 | |
| **Deafness** | | | | | | |
| Yes | 11 | 64.7 | 10 | 83.3 | 44 | 63.8 |
| No | 6 | 35.3 | 2 | 16.7 | 25 | 36.2 |
| NA[1] | 6 | | 5 | | 27 | |

| | | | | | | |
|---|---|---|---|---|---|---|
| **Cutaneous photosensitivity** | | | | | | |
| Yes | 16 | 69.6 | 14 | 93.3 | 48 | 78.7 |
| No | 7 | 30.4 | 1 | 6.7 | 23 | 21.3 |
| NA[1] | 0 | | 2 | | 25 | |
| **Dental anomalies** | | | | | | |
| Yes | 6 | 40 | 3 | 42.9 | 32 | 61.5 |
| No | 9 | 60 | 4 | 57.1 | 20 | 38.5 |
| NA[1] | 8 | | 10 | | 44 | |
| **Arthrogryposis** | | | | | | |
| Yes | 2 | 12.5 | 2 | 22.2 | 15 | 26.3 |
| No | 14 | 87.5 | 7 | 77.8 | 42 | 73.7 |
| NA[1] | 7 | | 8 | | 39 | |

**Suppl Table 3.** Subtype description by mutation position and type. 1: PAVs = protein altering variants; 2: PTVs = protein truncating variants; 3: 2 mutations upstream of PiggyBac; 4: 1 mutation upstream and 1 mutation downstream of PiggyBac; 5: 2 mutations downstream of PiggyBac.

| **b.** | | **2U[3]** | | **1U1D[4]** | | **2D[5]** | |
|---|---|---|---|---|---|---|---|
| | | **No.** | **%** | **No.** | **%** | **No.** | **%** |
| **PAVs[1]** | **Type II/COFS** | 0 | 0 | 0 | 0 | 24 | 64.9 |
| | **Type I** | 0 | 0 | 4 | 100 | 10 | 27 |
| | **Type III** | 0 | 0 | 0 | 0 | 3 | 8.1 |
| **PTVs[2]** | **Type II/COFS** | 9 | 39.1 | 5 | 38.5 | 31 | 56.4 |
| | **Type I** | 11 | 47.8 | 7 | 53.8 | 20 | 36.3 |
| | **Type III** | 3 | 13.1 | 1 | 7.7 | 4 | 7.3 |

**Suppl Table 4.** Multivariate ordinal logistic regressions odds estimates. Risk of having a more severe subtype depending on the position of the mutations and the type of variants. OR=odds ratio; CI=credibility intervals; Pr(OR>1)=probability that the OR is higher than 1 (%). 1: $OR_p$[CI]=odds ratio *a priori* with its credibility interval used to determine prior distribution for the model parameters.

| **Models** | **Priors N(μ, σ²)** | **Variables** | **OR [CI]; Pr(OR>1)** |
|---|---|---|---|
| 1 | Log(OR)~N(0,1000) | **Position of the mutations** | |
| | $OR_p$[CI][1] = 1 [0 – 8.3*$10^{26}$] | 1U1D | 0.9 [0.3 – 2.7]; 40.9 |
| | | 2D | 1.9 [0.8 – 4.7]; 92.2 |
| | | 2U | Reference |
| | Log(OR)~N(0,1000) | **Type of variants** | |
| | $OR_p$[CI][1] = 1 [0 – 8.3*$10^{26}$] | PTV | 0.9 [0.4 – 1.9]; 40.6 |
| | | PAV | Reference |
| 2 | Log(OR)~N(0, 0.674) | **Position of the mutations** | |
| | $OR_p$[CI][1] = 1 [0.2 – 5] | 1U1D | 0.9 [0.4 – 2.2]; 40.0 |
| | | 2D | 1.8 [0.9 – 3.8]; 94.7 |
| | | 2U | Reference |
| | Log(OR)~N(0.661, 0.234) | **Type of variants** | |
| | $OR_p$[CI][1] = 1.9 [0.75 – 5] | PTV | 1.2 [0.7 – 2.1]; 71.5 |
| | | PAV | Reference |
